# Successful Percutaneous Treatment of Left Main Artery Occlusion Associated With Focal Type A Aortic Dissection

**DOI:** 10.1016/j.jscai.2024.102293

**Published:** 2024-09-10

**Authors:** Ashraf Samhan, Anjan Tibrewala, Anirudh Kumar

**Affiliations:** a Department of Medicine, Feinberg School of Medicine, Northwestern University, Chicago, Illinois; b Division of Cardiology, Department of Medicine, Feinberg School of Medicine, Northwestern University, Chicago, Illinois; c Division of Cardiology, Department of Medicine, Central Dupage Hospital, Chicago, Illinois

**Keywords:** case report, dissection, left-sided catheterization, mechanical circulatory support, percutaneous coronary intervention

## Abstract

Type A aortic dissection is a rapidly progressive disease process with often fatal complications. We present a case of focal Type A aortic dissection complicated by left main occlusion and cardiogenic shock, treated with percutaneous coronary intervention and mechanical circulatory support.

## Introduction

Type A aortic dissection (TAAD) is a cardiovascular emergency classified as an intimal tear involving the ascending aorta. Prompt diagnosis is challenging as it presents similar to other life-threatening pathologies, including myocardial infarction and stroke. Common complications include aortic regurgitation and cardiac tamponade, which can lead to a 50% mortality rate within 48 hours.^1^ Rarely, the dissection can extend into the coronary arteries causing obstruction. Neri et al^2^ described 3 types of coronary lesions resulting from aortic dissection: (1) limited to the coronary ostium, (2) features a coronary false channel, and (3) circumferential detachment with intussusception. Surgical repair remains the gold standard; however, select patients are deemed unsuitable owing to advanced age and comorbidities.

## History of presentation

A 76-year-old woman presented to the emergency department with a several-hour history of substernal aching chest pain radiating to the back, found to have a non–ST-elevation myocardial infarction with an initial troponin of 138. Physical examination revealed no cardiac murmur or abnormal breath sounds. Six hours after admission, she developed worsening chest pain, hypotension, and acute hypoxic respiratory failure. Her blood pressure was 61/31 mm Hg, heart rate was 73 beats/min, and respiratory rate was 32 breaths/min on 3 L nasal cannula. A subsequent high-sensitivity troponin was 4230, prompting urgent left heart catheterization (LHC) overnight.

## Medical history

The patient’s history was significant for heart failure with recovered ejection fraction, hypertension, hyperlipidemia, tobacco use disorder (a 57 pack-year history), chronic kidney disease secondary to proliferative glomerulonephritis, hypothyroidism, plasma cell dyscrasia treated with 6 cycles of daratumumab/bortezomib/dexamethasone, and a recently treated left peroneal deep vein thrombus.

## Differential diagnosis

The differential diagnosis for her chest pain and hypoxia in the setting of a high troponin included acute coronary syndrome, aortic dissection, myocarditis, chemotherapy-induced cardiomyopathy, Takutsubo cardiomyopathy, acute valvular insufficiency, and massive pulmonary embolism.

## Investigations and management

Electrocardiogram on the floor revealed sinus bradycardia with nonspecific ST and T-wave abnormalities. Transthoracic echocardiogram revealed newly dilated and reduced left ventricular systolic function to 29%; severe hypokinesis of the entire anterior wall, mid-distal lateral wall, and entire apex; and moderate to severe mitral regurgitation. Laboratory tests were remarkable for a high-sensitivity troponin that peaked at 52,211 pg/mL and brain natriuretic peptide of 449 pg/mL.

**Figure 1:**
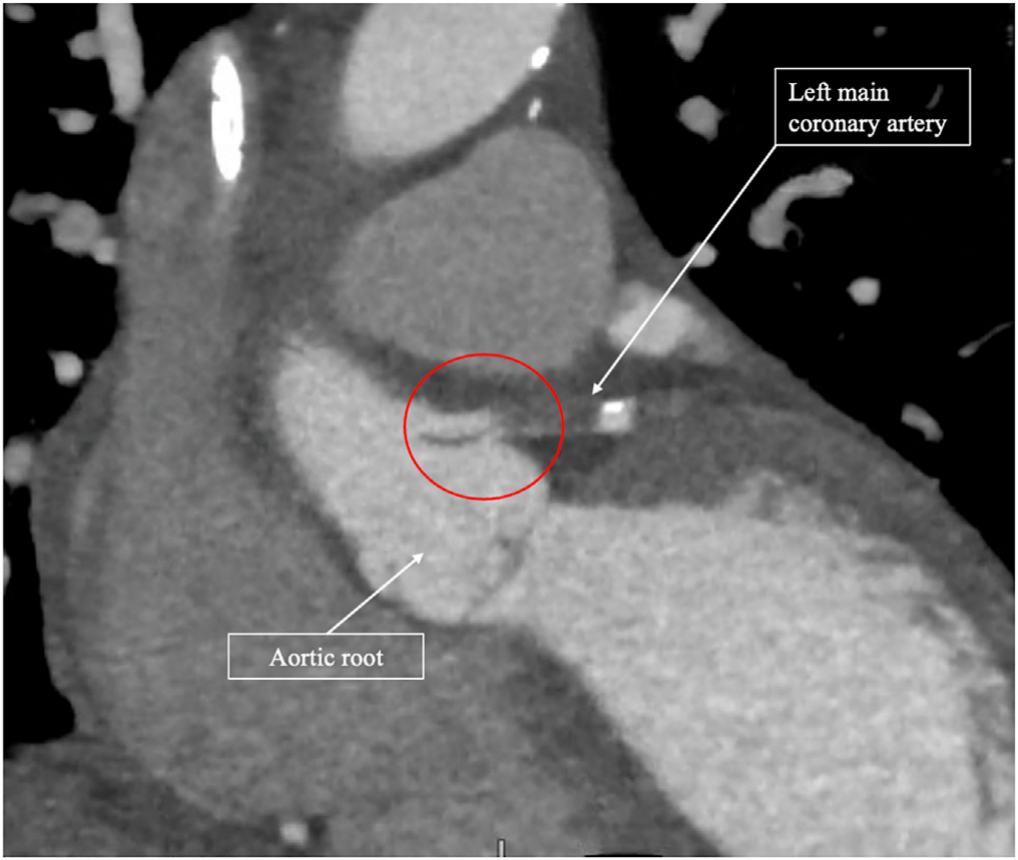
**CTA gated thoracic aorta revealing a type A aortic root dissection (red circle) extending into the left main coronary artery with subtotal occlusion.** CTA, computed tomography angiography.

Diagnostic LHC revealed a pulsatile compression of the ostial to distal left main (LM) artery concerning for a focal dissection with otherwise mildly diffuse coronary artery disease and severely elevated left ventricular end-diastolic pressure of 39 mm Hg (Supplemental Video 1). Given the concern for cardiogenic shock and critical LM disease, an intra-aortic balloon pump was placed to unload the left ventricle and improve coronary perfusion. Following the LHC, a computed tomography angiography–gated thoracic aorta was obtained to evaluate the extent of the dissection, which showed a focal aortic root dissection involving the LM and terminating in the sinotubular junction (Figure 1). There was no evidence of aortic arch involvement. These findings, in addition to her acute decompensation before invasive coronary angiography, supported the diagnosis of a spontaneous, rather than iatrogenic, focal TAAD causing coronary obstruction leading to non–ST-elevation myocardial infarction. Cardiothoracic surgery was consulted and deemed the patient a prohibitive surgical risk for open repair owing to an elevated risk of intraoperative mortality and multiple comorbidities, including advanced age, frailty, significant left ventricular dysfunction, oliguric acute kidney injury requiring continuous venovenous hemofiltration, and hemodynamic instability. Their recommendation was medical management and consideration for Impella-assisted percutaneous coronary intervention (PCI).

**Figure 2:**
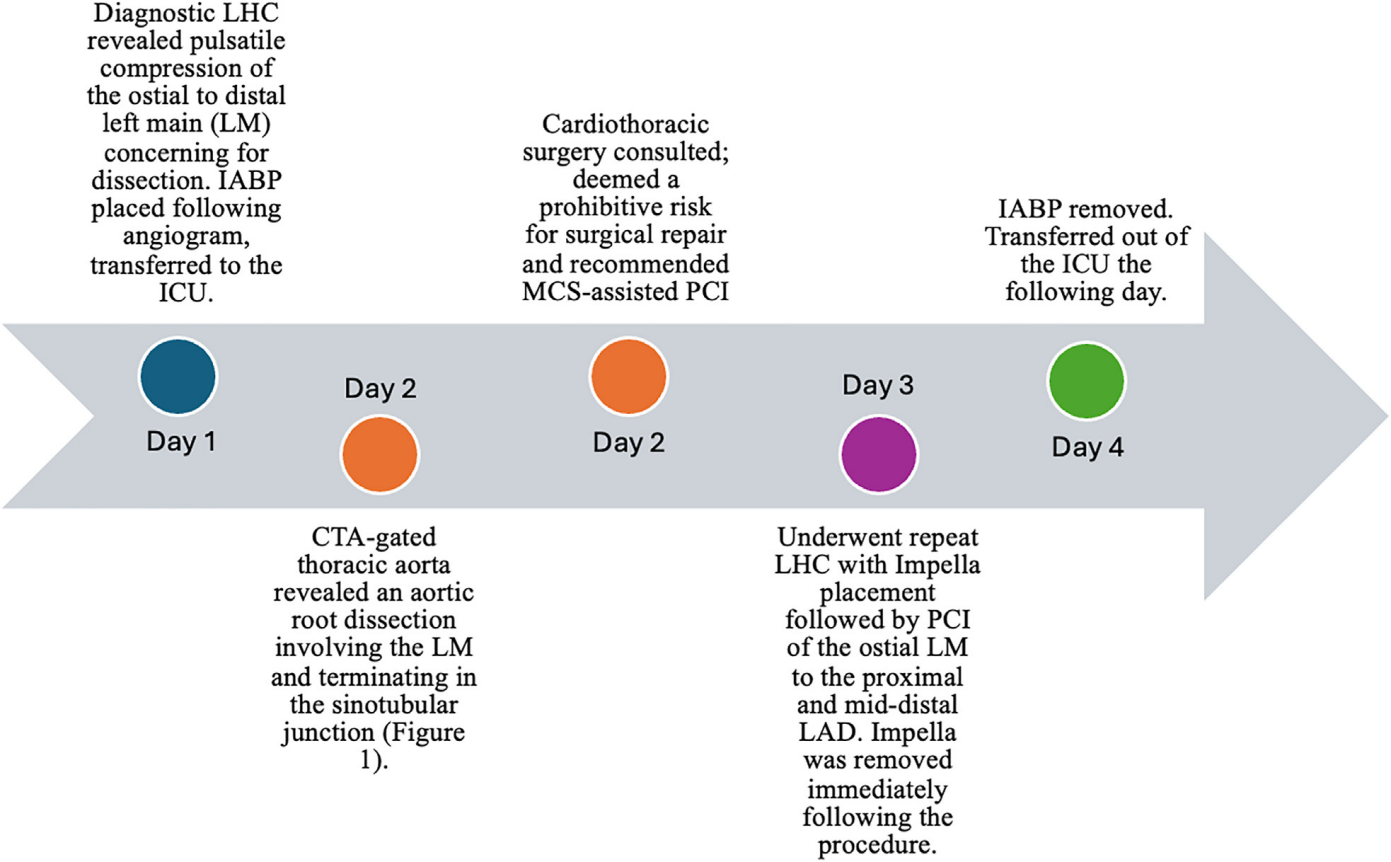
**Timeline of investigative and therapeutic interventions during hospitalization.** CTA, computed tomography angiography; IABP, intra-aortic balloon pump; ICU, intensive care unit; LAD, left anterior descending artery; LHC, left heart catheterization; MCS, mechanical circulatory support; PCI, percutaneous coronary intervention.

Two days after the initial diagnostic angiogram, the patient underwent PCI of the ostial LM to the proximal left anterior descending artery (LAD) in a provisional fashion with Impella CP support (Supplemental Videos 2 and 3). Extension of the dissection was noted in the mid-distal LAD, which was also stented. Intravascular ultrasound confirmed stent placement in the proximal LAD with no dissection distal to the stent edge and ostial coverage of the LM with a large hematoma (Supplemental Video 4). Final angiography showed no evidence of dissection or perforation. There was TIMI 3 flow and 0% residual stenosis visualized throughout. Following the procedure, the patient was able to successfully wean from mechanical circulatory support. Intra-aortic balloon pump was removed the following day, and the patient was transferred out of the intensive care unit 48 hours after PCI (Figure 2).

## Discussion

Acute aortic dissection is a challenging clinical emergency with diverse clinical features and lethal complications.^3^ Stanford type A dissection is defined as a tear between the media and intimal layers involving the ascending aorta proximal to the innominate artery. It can extend proximally or distally, commonly leading to cardiac tamponade, myocardial ischemia, and acute heart failure due to aortic regurgitation.^4^ Coronary malperfusion is an uncommon but fatal complication associated with a cumulative mortality rate of 1% to 2% per hour after symptom onset, with a higher incidence involving the right coronary artery.^5^ The gold standard for treatment is open surgical repair; however, this is not an option in cases of hemodynamiclity and severe comorbidities, leading to an in-hospital mortality rate of 58%.^3^

To our knowledge, this is 1 of the 2 reported cases of spontaneous focal TAAD resulting in LM occlusion leading to cardiogenic shock, which was successfully treated with PCI. Ravandi and Penny^6^ described a case of aortic dissection complicated by aortic insufficiency and total LM occlusion that was treated with PCI but the patient had known longstanding aortic root dilation. Several case reports have published TAADs presenting as acute myocardial infarction due to LM occlusion; however, all cases used PCI as a bridge to surgery and not definitive therapy.^5^^,^^7^, ^8^, ^9^, ^10^ Hence, clinicians should consider the possibility of PCI as a destination therapy for stenting the LM in patients who are poor surgical candidates.

This clinical case is unique because the aortic dissection was localized to the aortic root and extended into the LM artery, without involving the aortic arch or valve. While this type of dissection pattern is typically catheter induced, several aspects of the case support that the patient’s presentation was spontaneous due to an aortic dissection. First, she presented with chest pain radiating to the back as her initial complaint. Second, the patient had clinical decompensation and cardiogenic shock before the LHC. Given no other lesions identified on the angiogram, her symptoms cannot be attributed to anything other than the LM dissection. Third, the operator who performed the diagnostic angiogram never directly engaged the LM. Finally, it is unlikely that a patient who had untreated catheter-induced LM dissection would have remained stable until definitive treatment was performed.

### Follow-up

Serial echocardiograms revealed persistent severe left ventricular dysfunction with a left ventricle ejection fraction of 20% and mild-to-moderate mitral regurgitation. She was started on dual-antiplatelet therapy and low-dose metoprolol but was not been able to tolerate additional goal-directed medical therapy because of hypotension and bradycardia. She was seen in cardiology clinic 1 week after discharge and noted to be ambulatory without recurrent chest pain or dyspnea. A follow-up computed tomography angiography was ordered to serially monitor the aortic root intimal flap but has not yet been completed at the time of writing. The patient remains alive more than 4 months after PCI.

## Conclusion

Spontaneous aortic root dissection involving the LM is a rare cause of myocardial infarction. To our knowledge, this is a unique case that highlights focal TAAD resulting in LM occlusion that was successfully treated with PCI. In patients who are excluded from surgical repair owing to comorbid conditions, a percutaneous interventional approach can be considered as a beneficial alternative.

## Supplementary material

Supplementary Videos
