# Supplementary figures and images for: Successful Percutaneous Treatment of Left Main Artery Occlusion Associated With Focal Type A Aortic Dissection
Source: J Soc Cardiovasc Angiogr Interv. 2024 Sep 10;3(10):102293. doi: 10.1016/j.jscai.2024.102293 (PMC11549511; doi:10.1016/j.jscai.2024.102293)

## Slide 1
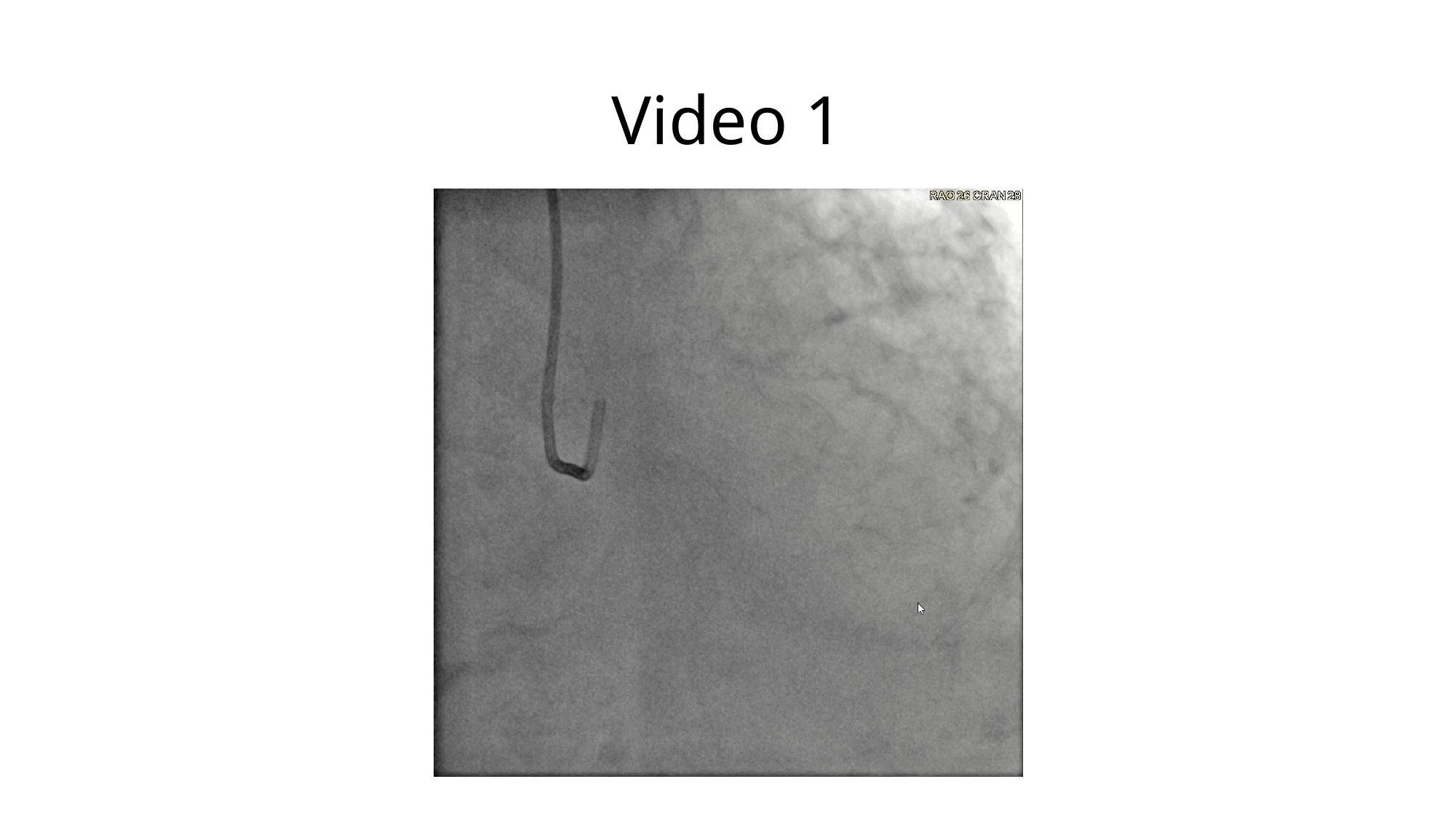

# Video 1

## Slide 2
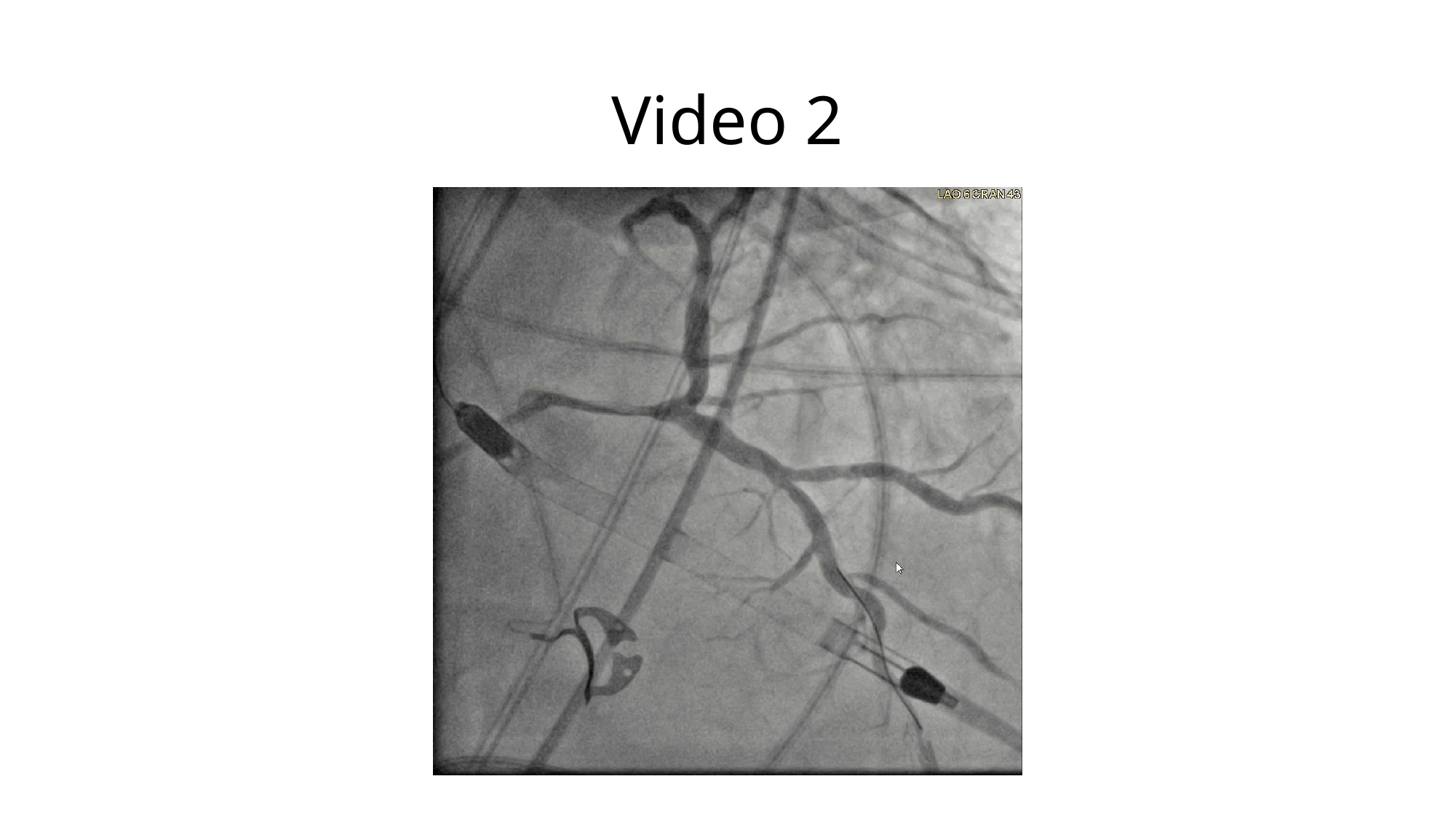

# Video 2

## Slide 3
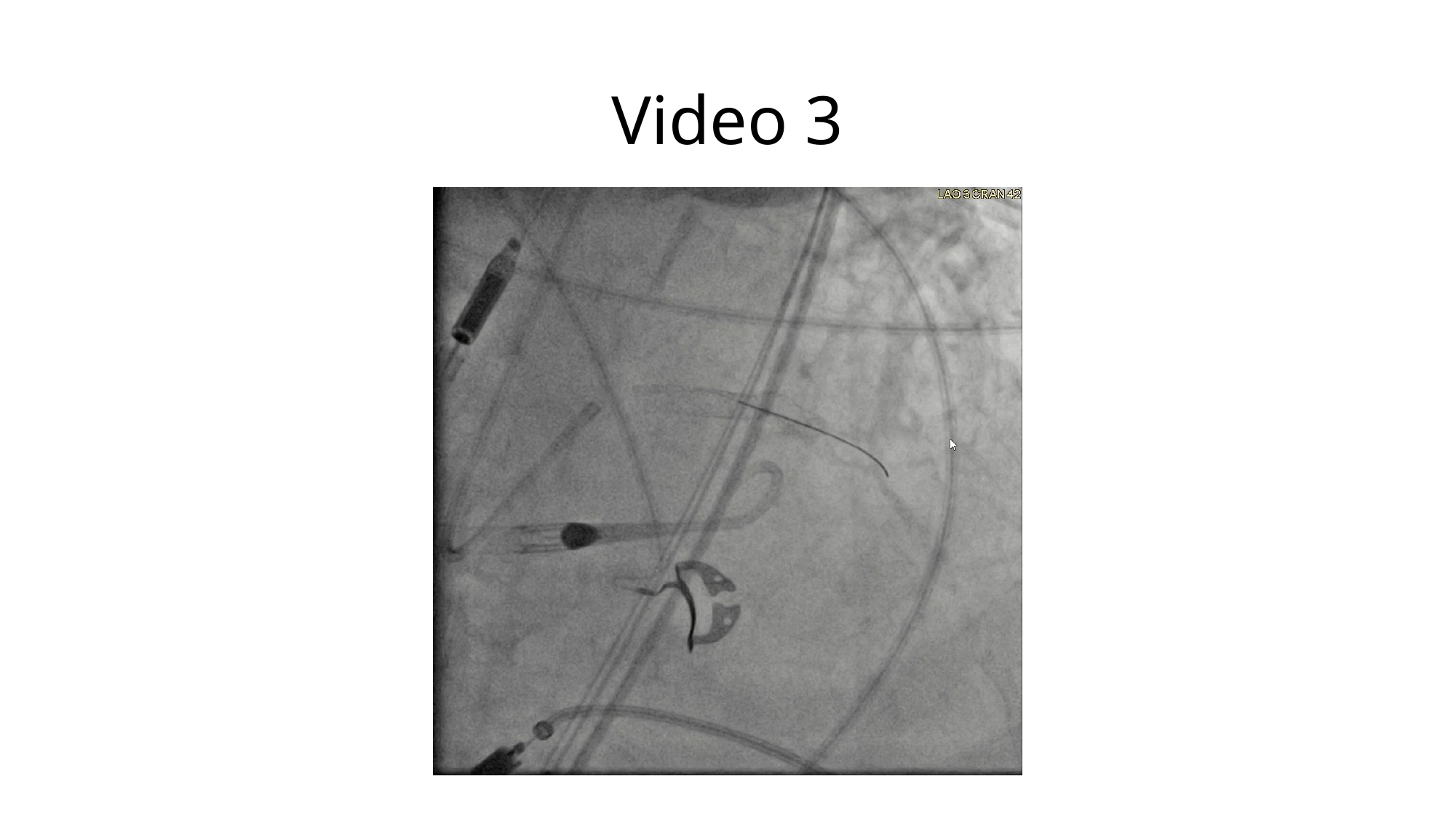

# Video 3

## Slide 4
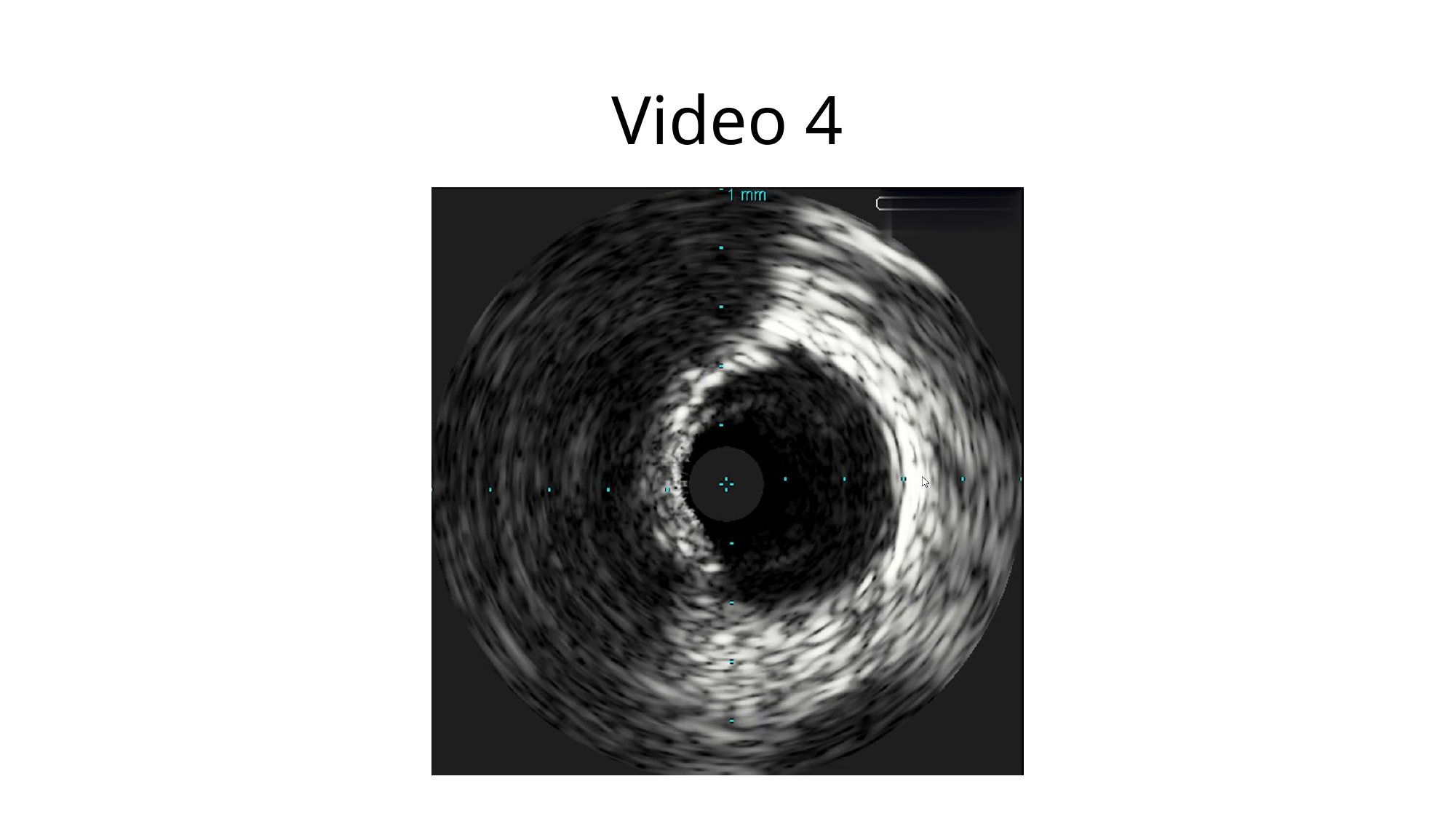

# Video 4

Supplement: Supplementary Videos [file mmc1.pptx]
